# Dr. Lilian M Abbo: Blazing trails and building a “global ID family” from Caracas to Miami

**DOI:** 10.1017/ash.2023.187

**Published:** 2023-06-19

**Authors:** Lilian M. Abbo

**Affiliations:** ^1^ Jackson Health System, Miami, Florida; ^2^ Division of Infectious Diseases and Miami Transplant Institute, Jackson Memorial Hospital, Miami, Florida

1. You’ve combined careers in infection prevention, stewardship, and transplant infectious diseases. How did that unfold, and what inspired you to pursue this unique career path?

I was born in Philadelphia, Pennsylvania. When I was 3 months old, we moved back to Caracas, Venezuela, where I went to medical school and lived until the year 2000. While completing a mandatory “rural medicine year,” I got married and emigrated to the United States with my husband.

From early in my medical training, I was exposed to the harsh reality of practicing medicine in a developing country where resources are extremely limited, antibiotics are prescribed over the counter, and self-medicating ailments with borrowed, leftover antibiotics is a common practice. Yes, my community was part of the problem of fueling antimicrobial resistance but also the likely reason I gravitated to a career in stewardship.

A good medical history and your clinical skills were often your only and most valuable tools. We had to make complex diagnoses and provide care to the very poor and critically ill with limited or no technology. Sometimes we paid out of our own pockets for our patients’ medications, sutures, and sterile gloves. The gloves were resterilized in the autoclave, and I don’t recall ever using a contact isolation gown during my training; we were grateful to have access to soap and clean water.

Some of the public health issues I encountered in Venezuela that inspired me to pursue a career in infectious diseases included campaigns to educate patients on the benefits of good hygiene and dehydration prevention (given that diarrhea was a leading cause of pediatric deaths), contraceptive care to limit adolescent pregnancies and the spread of HIV, and free immunization programs. We also had very strong training in microbiology, parasitology, and tropical medicine. I was driven to pursue infectious diseases (ID) by exposure to many cases of dengue, malaria, Chagas and later, HIV in the mid-late 1990s.

When I first came to the United States, I started working as an unpaid observer in the HIV/Special Immunology Division at Jackson Memorial Hospital. My first experience in the United States was with Dr. Luis Espinoza in the HIV division at the University of Miami. Luis invited me to bedside rounds and ultimately became a great friend, mentor, and sponsor early in my career. During my internship at Jacobi Medical Center in the Bronx, my best mentors were my peers and the second-year residents. Despite the challenging, resource-limited environment of a public hospital, which often reminded me of home, I still remember the positive and cheerful attitude of my cointerns like Bhaskar Gundabolu and Bredy Pierre-Louis, who kept reminding me that “each day has only 24 hours,” and that better days are coming soon.

During my internal medicine residency at the Mount Sinai Medical Center in Miami, Florida, I had an amazing clinical mentor, Dr. Kenneth Ratzan, an ID physician who inspired me to follow in his footsteps. He taught me that, to make a good ID diagnosis, “Go where the money is” (eg, get that lung biopsy) and to be fearless when advocating for the best patient care.

During my ID fellowship at the University of Miami and Jackson Memorial Hospital, I fell in love with the field of transplantation, which involved caring for the most complex patients with serious infections whose treatment is further complicated by multiple drug interactions. In organ donation, “Death becomes life.” We largely depend on deceased donors whose surviving family members are asked to donate their organs to honor their legacies. Thanks to that non-reciprocal gift of life, we can provide hope to thousands of patients. This concept, and multiple opportunities for growth, and innovation are what most drew me into transplantation infectious diseases (transplant ID).

Alongside talented colleagues, I helped care for many critically ill, immunocompromised patients within our underresourced system. Some of my early mentors were transplant surgeons like Dr. Gaetano Ciancio, who first introduced me to research in transplantation. I learned that keeping an open mind and reaching out to established and well-known individuals are extremely important, however intimidating. The first such person I contacted was Dr. Arjun Srinivasan from the CDC. I reached out as a brand-new attending physician asking to borrow a survey he published while at Hopkins. I was stunned when he replied to my email, shared his survey, and offered to help with my study. Since then, Arjun has been a great friend; I remain grateful for his national advocacy and leadership in antimicrobial stewardship and mentoring and sponsoring so many of us.

As I embarked on my journey in transplant ID, another major opportunity arose. Jackson Memorial Hospital offered me the position of Medical Director for the Antimicrobial Stewardship Program. I had to learn on the job but was blessed to have an amazing partner and friend as our stewardship pharmacist. For the next 10 years, Dr. Laura Aragon and I worked tirelessly to reinvigorate and expand the mission of stewardship across the health system. I had wonderful mentors, like Dr. Thomas (Mac) Hooton, who was instrumental to my success. He encouraged me to develop my first business plan and advocate for resources and effort commensurate to my workload. He also encouraged me to volunteer for IDSA and to get involved in national committees. He stressed the importance of collecting and analyzing data and publishing outcomes. At the time, I never would have imagined that I would eventually become the first Hispanic woman to serve on the IDSA Board of Directors!

In 2016, I was tasked with merging and overseeing the infection prevention and antimicrobial stewardship programs and expanding our work across a complex public health system. The day after I assumed the new role, the first case of Zika was reported in the United States. In hindsight, the spur-of-the-moment television interviews and press conferences on Zika prepared me well for the eventual COVID-19 pandemic.

As my colleagues and I developed a track record of success in transplant ID, stewardship, and infection prevention, the health system administration became more supportive of program growth. We learned to develop business proposals, to negotiate, and to allocate additional time and resources to expand the programs to what they are today. Our success can be attributed to a keen focus on identifying needs, gaps in knowledge, and opportunities for improvement. I believe that aspirational attributes of successful healthcare teams include relentless teamwork, passion, patient advocacy, fearless leadership, and a growth mindset. Merging a career in transplant ID, ASP, and IP has been an arduous process filled with challenges and successes; it has taken a village!

1. What lessons from transplant ID can be applied to IP and stewardship and vice versa? Is it difficult to be a good steward while caring for transplant patients? Moreover, do we need a specialized approach to HAI prevention in immunocompromised hosts, in your opinion?

I strongly believe that, to conduct effective antimicrobial stewardship, we need robust collaborations and integration with infection prevention. We can decrease, restrict, and discontinue antibiotics, but without a safe and healthy environment, basic preventive measures like hand hygiene, and appropriate cohorting and isolation protocols, antimicrobial resistance will continue to spread and our dependence on antibiotics will only increase. We also need to reframe cost effective “diagnostic stewardship”. With the explosion of molecular testing, genome sequencing and other rapid diagnostics, is key that we understand the value and analyze patient outcomes. This requires looking at things with a different lens and making decisions with “precision medicine”.

In transplant ID, this issue is increasingly important and complex. Although being a good steward is not difficult, a different kind of stewardship is necessary. Traditional stewardship goals such as cost savings and de-escalation need to be fine-tuned when applied to transplant ID. For instance, understanding when “shorter is better” can be applied to transplant patients is as important as appreciating when escalation or combination therapy is necessary. Finally, it is crucial to understand that you are part of a larger team and that transplant teams are a family, drama and all! Communication, team building, and picking your battles are key to thriving as a team with the unified goal of securing the best patient outcome.

I do believe we need a specialized approach to HAI prevention in transplant patients and a more thoughtful approach to characterizing HAIs. We are sometimes penalized based on criteria that are not suited to this population. Identifying evidence-based approaches to preventing and reporting HAIs is a major knowledge gap, and research is needed in both adult and pediatric transplant recipients. We also need to update the national IT interface of NHSN and make it robust and user friendly!

1. You’ve always embraced your Venezuelan roots and live authentically, which is very inspiring to others. How has this enhanced your relationship with your patients, mentees, and colleagues?

Being a Venezuelan Hispanic woman is central to my identity. It has enabled me to see life through music, dance, and a more colorful lens, even during difficult times. I am extremely grateful for the education and family values instilled in me in my youth and for my lifelong friends from back home. I cherish being able to communicate fluently with my Spanish speaking patients and understand their circumstances, social determinants of health, and cultural values affecting their care. I love serving as a role model for my trainees, research coordinators, and mentees from minority backgrounds. Living authentically and taking the road less traveled enables me to spread the hopeful message, “I did it and so can you.” I’m overjoyed to witness their success when provided with the right tools and opportunities. I like to refer my network of collaborators and mentees around the world as my “global ID family.”

1. What are the biggest internal drivers of your success? (Don’t be shy.)
  - Family values: My grandparents were holocaust survivors. I was raised to believe that no matter the circumstance, you can change your reality, you are not a victim, you can have it all in life (though not always at the same time).
  - The drive to help people: The opportunity to practice medicine is an honor and a privilege. Moreover, to have an impact at the health system level, nationally and internationally in infection control, hospital epidemiology, stewardship, transplant ID, and healthcare administration, is even more fulfilling.
  - There is no leadership without followers; my teams are filled with “A” players who strive for excellence and always go the extra mile.
  - Having amazing friends and a supportive network of collaborators.
  - I relentlessly pursue gender equity, leadership development, and career advancement opportunities for Hispanics and other minorities.
  - Never stop learning, being curious, leading by example and “walking the talk.”
  - My family (especially my kids): They inspire me to be the best version of myself and pay it forward.
2. How do you stay so well adjusted, positive, and full of energy? What’s your secret to always bringing your “A-game” to the professional space?

I love this question! Nobody is always 100% positive and full of energy, but I credit the following:

- Strong Coffee
- Gardening (helps me maintain my Zen)
- Working out (I love Zumba, kick boxing, yoga, and dancing in general)
- Traveling (exploring the world and different cultures feeds my soul)
- Creating boundaries (learning to say NO is hard but necessary)
- Gratitude. You only live once, but if you do it well, once is enough!

1. Describe a pivotal mentor relationship that altered the trajectory of your career

I’ve mentioned several important mentors already, but the first, most enduring, and important is my father. I come from a long line of physicians, yet I am the first female physician in my family. My father is a physician in Physical Medicine and Rehabilitation and a University Professor. He once said to me, “The satisfaction of helping another human, and the gratitude from alleviating their pain or suffering is greater than any amount of money.” If you follow your values, money will follow, not the other way around. That advice convinced me to pursue medicine as the path with the greatest opportunities to help others. Another pivotal mentor was my uncle, Dr Isaac Abadi. He was a brilliant physician, a pioneer in evidence-based medicine in Venezuela, and founder of the National Institute of Rheumatological Diseases in Caracas. My uncle taught me the value of a detailed medical history and a thorough physical exam, and the importance of applying analysis of the primary literature to patient care.

One of my dearest mentors is Dr. Ingrid Vasiliu-Feltes, a trailblazer in healthcare leadership, innovation, and administration. Ingrid recruited me as the Associate Chief for Patient Safety and Quality at the University of Miami Health System in 2014. She encouraged me to get my green belt certification in lean six-sigma, to become the president of Women in Academic Medicine at the University of Miami, and to start leadership development programs for women and other minorities. Finally, Kathleen Sposato, Dr. Rossana Rosa, and Dr. Ella Ariza, are 3 amazing women who have taught me to become a better listener, innovator, and mentor.

1. What were some notable challenges in your career and how did you address them?

Being a woman, a foreign medical graduate with no research mentorship and no connections within the US health system, I had to work as an observer for a year, prove my value, and adapt to a very different culture. Writing a personal statement about my accomplishments for residency applications was particularly difficult because I was unaccustomed to self-praise. Relearning medical English to write and publish manuscripts in high-impact journals was also challenging because all my training was in Spanish. Thus, I spent many sleepless nights editing and re-editing manuscripts with my esteemed mentor, Dr. Thomas Hooton.

Initially, I had no background in the business of medicine, how to negotiate a contract, how to develop a business plan, how to understand the concept of “return on investment,” nor how to pitch new ideas to hospital administration. I learned from my setbacks and discovered innovative ways to achieve more with less and to advocate and negotiate for resources for me and my teams. I recently completed a 2-year executive MBA to expand my financial prowess, become a more effective leader, and improve my operational and organizational skills. There are no barriers, only opportunities.

1. What career advice would you share with young professionals just starting out in IP, stewardship, transplant ID, or all of the above?
  - Networking! Collaborations are key throughout your career regardless of your path.
  - Understand the continuum of patient care, costs, and workflows. Be able to communicate with healthcare administrators to present your business case and allocate time and resources effectively.
  - Be a good listener and learn to be an effective communicator of medical information. (You might be in the media spotlight during the next pandemic.)
  - Innovate, learn, and adapt.
  - Be passionate and fearless.
  - Learn from your failures (there will be many) and celebrate your successes.
  - Mentors come in many varieties and at different stages of our careers, remain grateful to those who support you and hold the ladder for others coming after you.
  - Practice self-care; our careers are important, but our well-being, friends, and family matter even more.
  - Life is not a race, it’s a marathon. You’re only competing with the best version of yourself.
  - Always be a patient advocate!
2. Finally, which nonmedical book, essay, or podcast has been eye-opening for you that you’d recommend to our readers?
  - Books: *Man Searching for Meaning* by Victor Frankl and *You are a Badass* by Jen Sincero
  - Podcasts: *The Happiness Lab* (by Prof Lori Santos); a great one in Spanish *“El Taller de Vanessa Coppel.”*

